# ASD and ADHD Comorbidity: What Are We Talking About?

**DOI:** 10.3389/fpsyt.2022.837424

**Published:** 2022-02-28

**Authors:** Camille Hours, Christophe Recasens, Jean-Marc Baleyte

**Affiliations:** Service universitaire de Psychiatrie de l'Enfant et de l'Adolescent, Centre hospitalier Intercomunal de Créteil, Créteil, France

**Keywords:** autism, attention deficit and hyperactivity disorder, attention, agitation, restlessness, comorbidity, autism spectrum disorder

## Abstract

According to the scientific literature, 50 to 70% of individuals with autism spectrum disorder (ASD) also present with comorbid attention deficit hyperactivity disorder (ADHD). From a clinical perspective, this high rate of comorbidity is intriguing. What is the real significance of this dual diagnosis? Is ADHD in fact always present in such cases? Might the attentional impairment reported among our ASD patients actually be a distinct trait of their ASD—namely, impaired joint attention—rather than an ADHD attention deficit? Could their agitation be the consequence of this joint attention impairment or related to a physical restlessness etiologically very different from the agitation typical of ADHD? The neurobiological reality of ASD-ADHD comorbidity is a subject of debate, and amphetamine-based treatment can have paradoxical or undesirable effects in the ASD population. Consequently, does a dual diagnosis, notwithstanding its currency in the literature, prevent us from shedding sufficient light on major physiopathologic questions raised by the clinical picture of ASD?

## Introduction

The semiology of autism spectrum disorder (ASD) and attention deficit hyperactivity disorder (ADHD) presented in current nosography, which helps clinicians to identify these disorders, makes it clear that they are different entities, affecting children and their developmental histories in ways that are clearly distinct. In the first case, we are primarily describing distracted children who pay little attention in academic settings, lose their belongings, and have difficulty sustaining mental effort. In the second case, we are talking about children who seldom associate with others, have a hard time interacting and communicating, and may display unique motor or verbal behaviors, including stereotypies, echolalia, and idiosyncratic language. Whereas, children with ADHD tend to be relatively boisterous and talkative, and eager rather than apprehensive of interactions with peers or adults, autistic children may be distinguished by their repetitive and less coordinated motor function, difficulty communicating, emotions in sync with their sensory reality more than with their social setting, and uniform behaviors that keep the unpredictable at bay.

Epidemiologically, these two disorders also differ in their incidence. Their diagnoses are made at different ages. Children with ASD can be identified before they are 3 years old, while ADHD is diagnosed later on. Both fall into the wider category of neurodevelopmental disorders, within which “comorbidities” are considered relatively frequent.

The prevalence of ADHD in people with ASD ranges from 50 to 70%, according to the literature (1). Where does this figure come from? How was it obtained, which studies and semiological criteria were applied, and how were the relevant clinical data collected?

This figure is the product of meta-analyses. It is important to note the variability of findings between studies—more than a mean prevalence, a precise yet illusory value reflecting the view that this comorbidity is a clearly measurable entity—because it admits an alternative interpretation of the phenomenon in question. Indeed, reported rates of comorbidity range from 10 to 90%. In the logic of meta-analysis, variation is often explained through methodological arguments: studies diverging from the mean are said to describe different populations, apply less valid methods of measurement, or gather their data in atypical ways. Yet, in part, what may be described as “bias” could betray an unsound theoretical foundation, accommodating a multitude of experimental paradigms measuring what is thought to be the same phenomenon.

This article thus aims to recall the attentional features inherent to ASD; to describe and analyze the variability of data reported in the literature; and finally, to draw on studies of factors related to attention, such as memory, sensorimotor function, executive functioning, and intellectual disability, for a better understanding of the role these variables play in the expression of cognitive capacity within the ASD population.

## Autism and Attention

Attention is defined as a process of selection applied to the product of perception, and may even be directed toward memories. To direct attention on a perceptual or internally represented entity, our working memory must be trained on a goal for the duration of the task at hand. Attention encompasses essential elements of the executive functions and can be further broken down into sustained attention; focused attention; visual search, whereby the target of attention is defined using a template stored in working memory; voluntary or reflexive orienting and disengagement; attentional filtering; and expectation (2).

In people with ASD, these components of attention have a characteristic profile: sustained and focused attention is stronger than in normal subjects; visual search is also, though this seems to reflect the quality of perceptual processing more than attention; and the ability to orient attention toward non-social stimuli is deficient, as are reflexive and voluntary disengagement, in certain settings. Attentional filtering is not compromised in ASD patients having no intellectual disability. Higher-functioning ASD patients have a specific attentional impairment not observed among other ASD patients (3). Joint attention also appears to be lacking.

Can the comorbidity of ASD and ADHD truly be established by considering executive functions alone, as several studies suggested (4)? The developmental cognitive specificities of ASD have been well described as they are linked with perception aspects but their executive or attentional aspects have been relatively neglected. “Attentional impairments in autism tend to be more of the ‘not listening' and ‘difficulty shifting focus' type than of the ‘short attention span' and ‘excessive distractibility' type^1^.” These distinct clinical particulars suggest attentional symptoms inherent to ASD rather than comorbid ADHD.

## What Do Comorbidity Studies Address?

While the DSM-4 and ICD-10 give mutually exclusive diagnoses of ADHD and ASD, the DSM-5 mentions each condition in its description of the other, admitting the possibility of comorbidity.

Sprenger et al. (5) concluded that autistic symptoms were significantly more severe, especially in the area of social interaction (as evaluated by the social responsiveness scale and autism diagnostic interview), in patients with dual ASD-ADHD diagnoses than in those with ASD alone. Yet this conclusion might also illustrate the frequent clinical confusion of these disorders: could the severe autism they describe not just as well itself be the cause of a more symptomatic attention deficit, without suggesting the presence of ADHD? Similarly, Green et al. (6) stated that autistic symptoms were more prevalent in children with ADHD. Their study considered a group of children aged 6 to 10 divided into an ADHD subgroup and a control subgroup without ADHD. It seems questionable to conclude that autistic symptoms are more prevalent in ADHD patients without recognizing that the severity of ASD is independently at the origin of pronounced attentional deficiencies. The study also concluded that the intensity of hyperactive and impulsive symptoms directly impacts the severity of ASD symptoms, without inversely considering that, again, the severity of ASD might independently explain signs of psychomotor agitation and attentional deficiencies. Furthermore, findings did not differ by ADHD subtype, which further supports the hypothesis that the observed attentional deficit and motor hyperactivity are more directly explained by severe autism than comorbid ADHD.

Some studies have reported structural differences in attentional functions between ASD and ADHD patients, while others suggest these disorders present identical deficiencies (7, 8).

Barnard-Brak (9) reported varying ability to distinguish individuals with ASD from those with ADHD on the basis of their performance on different cognitive tasks that assess sustained attention. However, the rapid letter naming task, which is thought to predict surface reading ability and other reading skills, did reveal significant differences between ASD and ADHD children: the former spent more time on the task and performed better. Thus, the interpretation of performance on cognitive-attentional tasks evaluating sustained attention requires special caution to avoid confusing the two disorders. This study also highlighted the effect of environment on the performance of attentional tasks by those with ASD: the setting under which individuals complete diagnostic tests can greatly influence results.

Hochhauser et al. (10) described specific attentional traits related to social interaction in young adults with ASD that may, however, be the consequences of another form of cognitive impairment. Several studies have reported cognitive characteristics affecting attentional skills in the ASD population, including difficulty disengaging, significantly greater processing of local details, or heightened perception yet “context blindness”. These elements in turn influence processing speed. Processing speed is therefore not impaired directly, but rather, differences in how perceptual data are processed have an impact on attention. Hence, it would seem more appropriate to speak of attentional traits of ASD, rather than attentional anomalies or deficits, thereby distinguishing them from ADHD.

Mayes et al. (11) demonstrated that disruptive mood dysregulation disorder was extremely prevalent among children with ASD, significantly more so than among ADHD and neurotypical children. Moreover, 91% of the children with disruptive mood dysregulation disorder symptoms also satisfied criteria for oppositional defiant disorder, revealing the very high prevalence of externalizing behaviors in ASD. The presence of psychomotor agitation cannot be automatically attributed to ADHD motor hyperactivity but does suggest an emotional dysregulation disorder more directly linked to the behavioral effects of irritability.

## Physiopathologic Aspects: Imaging and Eeg Data

Is observed agitation then a sign of the prefrontal inhibitory deficit underlying ADHD or rather a state of restlessness, a minimal expression of cerebellar dysfunction, characterizing an etiologically distinct entity? The association of ASD with alterations in specific brain regions is becoming increasingly clearer. The affected regions include the orbitofrontal cortex, superior temporal sulcus, fusiform gyrus, amygdala, and cerebellum (12), and the latter plays a role in learning processes, memorization, several executive functions, and cognition. In light of these facts, might it not be risky to diagnose ADHD, a disorder linked with an altered prefrontal cortex, in the ASD population?

ADHD and ASD are described as frequently co-occurring, sharing certain cognitive phenotypes. However, it is important to be able to trace these shared features back to a common physiopathology and identify the physiopathologic characteristics of comorbidity, which may present additional neurofunctional deficits. Chantiluke et al. (13) compared prefrontal function in four groups of youth with ASD, ADHD, comorbid ASD and ADHD, or neither disorder (controls) through a temporal discounting task, using fMRI. They revealed anomalies shared by the non-control groups, in addition to distinct features unique to each of these three groups. In comparison with the non-comorbid and control groups, the comorbid group presented unique and more severe impairments affecting the lateral and ventromedial prefrontal cortex, ventral striatum, and anterior cingulate cortex. These physiopathologic findings suggest that ASD-ADHD comorbidity does not correspond to a mere combination or addition of both disorders: it is neurofunctionally distinct and merits further study for more accurate characterization.

As shown by Lau-Zhu et al. (14), ASD and ADHD are each associated with unique attention processing traits. Studies of event-related potentials (ERPs)—concerned with inhibitory control and performance monitoring in ADHD (15–17), and social or emotional processing as well as executive functioning in ASD (18)—mostly involving adolescents have reported distinct abnormal cognitive profiles for ADHD and ASD. Both disorders are associated with atypical allocation of attentional resources and atypical performance monitoring. However, the structural impairments underlying them are very different. With regards to attention, ADHD impairment tends to reflect difficulty detecting clues that would otherwise enable anticipation, while ASD impairment is more directly related to a heightened perceptual capacity and weaker orientation toward new inputs, with longer retention of stimuli in working memory and unique social, emotional, and executive functioning features. ADHD, unlike ASD, is more immediately linked to impaired inhibition. It is important to recall that impaired inhibition, the root physiopathologic feature of ADHD, has not been studied in cohorts of ASD patients. Sensory processing impairments, such as those observed in ASD, ultimately have repercussions on attentional processes. The causes of the attentional deficiencies seen in these two disorders would thus appear to be very distinct.

Research applying quantitative EEG has demonstrated atypical profiles for ADHD, principally concerning theta and beta frequency bands (17, 19), and for ASD, mainly related to alpha, beta, and gamma frequency bands (20). ASD and ADHD have distinct and overlapping features in “four neurocognitive domains: attention processing, performance monitoring, face processing and sensory processing” (14). Yet studies comparing the two disorders or considering dual diagnoses have yet to be undertaken. Further investigation into the neural bases of co-occurring ADHD and ASD would be of particular interest.

To better understand the distinct neuropsychological profiles of ADHD and ASD patients, it seems appropriate to consider visuospatial exploration strategies. In terms of visuospatial abilities, ADHD patients in a study by Cardillo et al. (21) exhibited a heterogeneous profile with more severely impaired visuospatial processing speed, while ASD patients and typically developing subjects had similar profiles. The authors also state that the local-global processing index effectively distinguishes these groups on the basis of performance on visuo-constructive tasks. Accordingly, a more detailed understanding of the neurocognitive specificities of each disorder might be acquired by accounting for the various domains of visuospatial processing.

## Discussion

It has often been stated in the literature that ASD and ADHD are difficult to distinguish when making a diagnosis. Mayes et al. (22), considering 847 children with ASD and 158 with ADHD, report that ADHD symptoms were commonly observed in ASD youth. Ratings of attention deficit, impulsivity, and hyperactivity were no different between children with ASD of any severity and children with ADHD-Combined type. Autism is very distinct from ADHD, but the core symptoms of ADHD-Combined type, i.e., attention deficit, impulsivity, and hyperactivity, would appear to also be features of autism. ASD and ADHD are neurobiological disorders characterized by similar underlying neuropsychological “deficits”. A similar observation is made by Van der Meer et al. (23). According to the authors, ASD and ADHD are different manifestations of one overarching disorder. They made the hypothesis there is a single continuum in which emotion regulation is a crucial common factor. In the same way, Ghirardi et al. (24) demonstrated there do exist a genetic overlap between clinical ASD and ADHD, suggesting an underestimation of this overlap by genomic studies. Van der Meer et al. also added that children with a ADHD phenotype without ASD symptoms can clearly be identified while the opposite is not true. These observations corroborated the assumption attention disorder is an inherent feature of ASD. Though Mayes et al. quickly conclude that “attention deficits” in ASD and ADHD are similar, they do report an interesting difference between ASD and ADHD children in this domain: selective attention is significantly more common among children with ASD (98%), no matter the severity of their disorder, than among those with ADHD-Inattentive type or ADHD-Combined type (21%). Whereas, children with ADHD have difficulty fixing their attention on a given task, those with ASD do have the capacity to focus on activities that interest them, e.g., puzzle assembly, reading, or repetitive drawing. Consequently, the authors reach the conclusion that ASD and ADHD can be distinguished by certain symptoms that differ considerably. While autistic symptoms are rarely seen in ADHD, there are specific ADHD symptoms that are particularly common in autism. Accordingly, we maintain that the definition of autism must take into account those symptoms that mirror or overlap with symptoms of ADHD, thereby better representing both the clinical reality of ASD, whose symptoms vary in intensity along a spectrum, and its neurobiological reality, i.e., the cortical dysfunction of which the clinical symptoms are an expression.

Mayes et al. also found no significant difference between children of normal intelligence with ASD, ADHD-Combined type, or ASD-Inattentive type in terms of performance on neuropsychological tests that evaluated attention, working memory, processing speed, and graphomotor skills.

Can comorbidity of ASD and ADHD truly be established by considering executive functions alone, as suggested by some studies (3)? Carter Leno et al. (25) reported that multiple executive functioning impairments typically associated with ADHD are also found in people with ASD. Neuropsychological evaluations of executive functions do not fully account for the complexity of symptoms, and broader studies that consider additional brain functions could provide more clinical data essential for diagnoses. The study by Carter Leno et al. emphasized the limits of executive function exploration for discriminating ASD, ADHD, and oppositional defiant disorder subgroups. The authors evaluated the performance of four groups of youth ages 10 to 16—a typically developing group (*N* = 43) and three groups of individuals clinically diagnosed with ADHD (*N* = 21), oppositional defiant disorder (*N* = 26), and ASD (*N* = 41), respectively—on go-no-go and switch tasks, and detected deficits shared by the ADHD, oppositional defiant disorder, and ASD groups, such as increased reaction time variability. After controlling for symptoms of ADHD and oppositional defiant disorder, differences in reaction time variability between groups were no longer significant. For cognitive flexibility, as evaluated by the switch task, there was also no observed difference between groups. The ASD group alone exhibited impaired response inhibition and premature responsiveness, relative to the typically developing group. Carter Leno et al. thus concluded that executive functioning impairments specifically described as present in ADHD are also found in ASD. This stresses the need to explore and precisely define the characteristics of attentional impairment in autism. These characteristics, their causes, and their consequences should be included in the definition of ASD, to more accurately represent its specificities.

These findings are supported by those of Rosello et al. (26), who reported significantly more ADHD symptoms and poorer learning behaviors in ASD children than in those exhibiting typical development. Furthermore, behavioral regulation problems and impaired executive functioning associated with ADHD symptoms significantly impacted performance for ASD children, objectively demonstrating the effect of ADHD symptoms on these children's learning behaviors.

The attentional impairment best described for ASD is diminished joint attention. It is also said to give rise to later social communication impairments, especially in connection with oromotor skills (27–29). Recent functional neuroimaging studies reveal the influence of neural mechanisms through which sensory processing and attention may be modulated by the affective impact of a stimulus. The amygdala plays a central role in the production of direct and indirect top-down signals along sensory pathways, shaping how emotional events are represented. “These modulatory effects implement specialized mechanisms of ‘emotional attention' that might supplement but also compete with other sources of top-down control on perception (30).” When interpreting attentional abilities, it therefore seems necessary to account for the role of neural processes and temporo-spatial dynamics of the brain determining how cognitive and affective elements are integrated into attention and behavior. These factors are fundamental to the study of attentional abilities in ASD patients in whom amygdalar dysfunction has been described. We may refer to the findings of Liss et al. (31), who demonstrated that sensory overreactivity is associated with overselective, hyperreactive and overfocused attention; perseverative and stereotyped behaviors; and excellent memory skills—but also with major social deficits. Sensory-seeking behaviors are strongly linked to overfocused attention. This kind of excessive attention can be mistaken for ADHD inattention.

With respect to psychomotor agitation, it has been posited that ASD patients have atypical arousal systems, their state of overexcitement reflecting both excessive and fluctuating cortical activation by the brain stem. Dopaminergic hyperactivity along the nigrostriatal (explaining stereotypies) and mesolimbic (explaining interpersonal and perceptual deficits) pathways is a hypothesis gaining wider support. It would explain the perceived effectiveness of dopamine antagonists, vs. agonists, on all symptoms of autism. The above confirms the need to include the characteristics of hyperfocus among the diagnostic criteria for autism. Hyperfocus is a dimension also suggested by MEG studies (32) pointing to long-range underconnectivity in ASD patients.

Mundy et al. (33) explain that joint attention plays a key role in the functional development of a distributed cortical system involving both anterior (prefrontal and insular cortices) and posterior (temporal and parietal cortices) neural networks. Hence, early impairment of joint attention has direct repercussions on all aspects of intero- and exteroceptive data integration, altering cortical processing. Interestingly, ASD patients are described as being slow to orient their attention. Harris et al. (34) show, in children with ASD, that delay in orienting attention to visual stimuli correlates with severity of cerebellar hypoplasia, as evaluated by MRI. No correlations were found with the sizes of other brain regions. When interpreting the unique cognitive traits of people with ASD, particular attention must be paid to the role of the cerebellum. This may afford a broader understanding of the neurocognitive processes involved in ASD and a clinical perspective that better reflects the neurobiological facts.

## Conclusion

To date, the mixed findings of etiologic ASD-ADHD comorbidity studies do not permit a clinical description of the physiopathologic comorbidity of these disorders. When speaking of comorbidity, are we not rather describing the severity of an attentional trait, with disabling functional effects, present in all ASD children?

These studies themselves demonstrate that children diagnosed with a comorbid disorder have more severe ASD. This suggests that—rather than an ASD-ADHD comorbidity, as authors conclude—the causes and consequences of a major attentional deficit typical of ASD are responsible for these patients' profiles. If so, this attentional trait should be included in the clinical definition and description of ASD. Instead of an ADHD explanation, we might then adopt an alternative etiologic perspective on these neurocognitive peculiarities and more fully account for the various brain impairments observed. As we have shown, children with ASD are affected much more by heightened attentional abilities than by a primary attention deficit, although the latter may be mistakenly (and dangerously) suggested by their clinical presentation. Therefore, we highlight the urgent need to develop new clinical and electrophysiological instruments to best fractionate and analyze these important neuropsychological features. This would be of crucial interest in particular for those modeling aspects of the co-morbidity in IPSC lines or animal models but also for the optimization of treatments use.

The attentional specificities observed in ASD, and their consequences, which are a direct reflection of unique brain functioning again challenge the validity of polythetic diagnoses in psychiatry. Attention deficits are key behavioral phenotypes of a considerable number of neurological and genetic diseases characterized by complex psychiatric disorders. Might the error lie in conflating such deficits with the unique, and very different, attentional traits of autism? In other words, are the attentional characteristics of disorders ignored by making an erroneous generalization?

Though further description of these characteristics is necessary, are they not key clinical symptoms of ASD in their own right—justifying their inclusion in the definition of this disorder—and potentially sufficient for its diagnosis?

## Data Availability Statement

The original contributions presented in the study are included in the article/supplementary material, further inquiries can be directed to the corresponding author.

## Author Contributions

All authors listed have made a substantial, direct, and intellectual contribution to the work and approved it for publication.

## Conflict of Interest

The authors declare that the research was conducted in the absence of any commercial or financial relationships that could be construed as a potential conflict of interest.

## Publisher's Note

All claims expressed in this article are solely those of the authors and do not necessarily represent those of their affiliated organizations, or those of the publisher, the editors and the reviewers. Any product that may be evaluated in this article, or claim that may be made by its manufacturer, is not guaranteed or endorsed by the publisher.

## Footnotes

^1^https://caddra.ca/pdfs/caddraGuidelines2011Chapter02.pdf.

## Abbreviations

ADHD: attention deficit hyperactivity disorder
ASD: autism spectrum disorder.
